# Mechanisms and Interventions of Diabetic Wound Healing

**DOI:** 10.2174/0115733998356905250706203510

**Published:** 2025-07-23

**Authors:** Juan Feng, Sien Lai, Dongsheng Tang

**Affiliations:** 1 Guangdong Provincial Engineering and Technology Research Center for Gene Editing, School of Medicine, Foshan University, Foshan, 528000, China

**Keywords:** Diabetic wound healing, cellular dysfunction, altered inflammatory responses, oxidative stress, glycation end-products, diabetic wounds

## Abstract

Diabetic wounds constitute a significant global health challenge, affecting millions of individuals worldwide and imposing a substantial burden on healthcare systems. This review explores the complex pathophysiology of diabetic wound healing and discusses innovative interventions aimed at addressing this critical clinical problem. The impaired healing process in diabetic wounds is characterized by a multitude of interrelated factors, including cellular dysfunction, altered inflammatory responses, oxidative stress, the formation of advanced glycation end-products, and neurovascular abnormalities. Fibroblasts, keratinocytes, and endothelial cells demonstrate diminished proliferation and migration capabilities, while immune cells exhibit dysregulated responses, which contribute to a persistent inflammatory state. Complications associated with diabetes, such as neuropathy and vascular insufficiency, further exacerbate the wound healing process. Recent advancements in wound care strategies have opened new avenues for enhancing diabetic wound healing. These advancements encompass the development of advanced dressings and biomaterials, growth factor therapies, cell-based interventions, and gene therapy approaches. The integration of diverse treatment modalities, coupled with the management of systemic metabolic abnormalities, offers significant promise for improving outcomes in diabetic wound care. Future research should focus on optimizing combination therapies, developing personalized treatment algorithms, and conducting large-scale clinical trials to establish the most effective and safest interventions for diabetic wound healing.

## 1 INTRODUCTION

Diabetic wounds constitute a major global health challenge, impacting millions of individuals worldwide and imposing a substantial burden on healthcare systems [1]. These chronic, non-healing wounds are a common and severe complication of diabetes mellitus, with a lifetime incidence of 15-25% among diabetic patients [2]. The global prevalence of diabetic foot ulcers is estimated at 6.3%, with variations observed across different regions and populations [3]. The impact of diabetic wounds extends beyond economic burden and physical discomfort, significantly diminishing patients' quality of life and increasing the risk of lower extremity amputations [4].

The healing of diabetic wounds presents distinct challenges due to the complex interplay of factors associated with the diabetic condition. These factors encompass impaired cellular and molecular mechanisms, altered inflammatory responses, increased oxidative stress, and the presence of advanced glycation end-products (AGEs) [5]. Additionally, diabetes-related complications, such as neuropathy and vascular insufficiency further exacerbate the wound healing process, creating a detrimental environment for tissue repair and regeneration [6].

Recent advancements in molecular biology, biomaterials science, and regenerative medicine have elucidated novel pathways and potential therapeutic targets [7]. These insights have facilitated the development of innovative approaches to enhance diabetic wound healing, including advanced dressings, and growth factor therapies, cell-based interventions, and nanotechnology applications [8]. Improved wound care strategies hold the potential to reduce healing time, decrease complication rates, and ultimately improve patient outcomes [9]. Furthermore, the effective management of diabetic wounds has the potential to substantially reduce healthcare expenditures and mitigate the socioeconomic burden associated with this chronic condition [10]. Consequently, it is important to understand the mechanisms and interventional strategies for diabetic wound healing.

This review aims to provide a comprehensive overview of the current understanding of the mechanisms underlying diabetic wound healing and explore innovative interventions that show promise in addressing this complex clinical challenge. By integrating insights from fundamental scientific research with emerging clinical approaches, we aim to highlight potential avenues for improving diabetic wound care and to identify areas for future investigation.

## 2 METHODOLOGY

We performed a comprehensive literature search using the PubMed database. The following search terms were used in various combinations: “diabetic wound”, “diabetic foot ulcer”, “wound healing”, “diabetes mellitus”, “therapy”, “treatment”, “mechanism”, “pathophysiology”, “intervention”, “biomaterials”, “growth factor”, “cell therapy”, “gene therapy”, “advanced dressing”, and “clinical trial”. The inclusion criteria were (1): original research articles, systematic reviews, meta-analyses, and clinical trials (2); studies focusing on mechanisms or interventions of diabetic wound healing (3); publications in English (4); studies with clearly defined outcomes related to wound healing (5); *in vivo* or clinical studies. The exclusion criteria were (1): case reports and conference abstracts (2); studies not focused on diabetic wounds (3); publications not in English (4); studies with incomplete data or unclear methodology.

## 3 NORMAL WOUND HEALING PROCESS

Wound healing is a complex and dynamic process characterized by a series of coordinated events, which can be broadly categorized into four overlapping phases: hemostasis, inflammation, proliferation, and remodeling [11]. The progression through these phases can be influenced by various factors, including age, nutrition, wound characteristics, and comorbidities such as diabetes [12].

The hemostasis phase begins immediately following injury, with the primary objective of arresting bleeding. This phase encompasses the processes of vasoconstriction, platelet aggregation and degranulation, and the formation of a fibrin clot [13]. The formation of the fibrin clot not only stops bleeding but also provides a provisional matrix that facilitates cell migration during subsequent wound healing phases [14].

The inflammatory phase is characterized by increased vascular permeability, the influx of immune cells, and the release of pro-inflammatory cytokines. These processes serve to clear debris and bacteria from the wound site and prepare for repair [15]. Neutrophils are the initial inflammatory cells to arrive at the wound site, followed by macrophages, which play a pivotal role in the transition from the inflammatory to the proliferative phase by producing growth factors such as platelet-derived growth factor (PDGF) and transforming growth factor beta (TGF-β) [16].

The proliferation phase encompasses key events, including angiogenesis, fibroplasia, re-epithelialization, and wound contraction, all of which are essential for filling the wound defect and restoring tissue integrity [17]. During this phase, fibroblasts assume a pivotal role by synthesizing new extracellular matrix components, especially collagen synthesis. Angiogenesis is stimulated by factors such as vascular endothelial growth factor (VEGF), which is produced by macrophages, keratinocytes, and endothelial cells in response to hypoxic conditions [18].

The remodeling phase involves the reorganization of collagen fibers, replacement of type III collagen with the more robust type I collagen, and a gradual reduction of wound vascularity, resulting in the formation of mature scar tissue [19]. This remodeling process is meticulously regulated by a balance between matrix metalloproteinases (MMPs) and tissue inhibitors of matrix metalloproteinases (TIMPs), which modulate the degradation and deposition of extracellular matrix components [20].

## 4 PATHOLOGICAL MECHANISM OF DIABETIC WOUND HEALING

The pathophysiology of diabetic wound healing is complex and multifaceted, involving numerous interrelated factors that contribute to impaired tissue repair. A comprehensive understanding of these mechanisms is crucial for the development of effective interventions and for enhancing patient outcomes (Fig. **1**).

### 4.1 Impaired Cellular and Molecular Mechanisms

In diabetic wounds, the cellular and molecular mechanisms critical for normal wound healing are disrupted. Fibroblasts, keratinocytes, and endothelial cells exhibit reduced or impaired proliferation, migration, and functionality [21, 22]. Specifically, keratinocytes display altered migration and differentiation, which compromises re-epithelialization [23]. These cellular dysfunctions are partially attributed to the effects of sustained hyperglycemia, which alters the microenvironment and cellular functions [24, 25].

#### 4.1.1 Fibroblasts

Fibroblasts play a crucial role in wound healing by synthesizing extracellular matrix (ECM) components, producing growth factors, and facilitating wound contraction. The repair of the dermis is primarily accomplished through the proliferation, differentiation, and ECM production of fibroblasts [26]. Additionally, fibroblasts can differentiate into myofibroblasts, which promote wound contraction and secrete proteases and collagen, contributing to the formation of granulation tissue [27]. However, the hyperglycemic environment and AGEs impair fibroblast function. In diabetic wounds, fibroblasts exhibit decreased proliferation rates and inhibited migration capabilities, which lead to impaired dermal repair and significantly impede the wound healing process [28]. Fibroblasts in a hyperglycemic environment show impaired collagen synthesis, leading to weakened ECM formation [5, 21]. In addition, diabetic fibroblasts show diminished responsiveness to growth factors such as PDGF and TGF-β, which are crucial for normal wound healing [24]. Fibroblasts from diabetic wounds often exhibit features of cellular senescence, including reduced proliferative ability and increased expression of senescence-associated β-galactosidase [29]. Senescent fibroblasts and macrophages can impede the process of diabetic wound healing through the CXCL2-CXCR2 signaling pathway [29, 30].

#### 4.1.2 Keratinocytes

Keratinocytes are essential for re-epithelialization and barrier restoration. In the early phase of wound repair, keratinocytes have the ability to directly eliminate invasive pathogens by secreting antimicrobial peptides, chemokines, cytokines, and extracellular vesicles to facilitate the interaction between keratinocytes and immune cells [31]. Keratinocytes migrate to the wound bed, proliferate, and differentiate into different structures to restore the integrity of the epidermis during wound epithelialization [27]. However, high glucose impairs the normal function of keratinocytes in diabetic wounds. In diabetic wounds, keratinocytes show reduced migratory capacity, partly due to decreased expression of integrins and alterations in the actin cytoskeleton [32]. The normal differentiation process of keratinocytes is disrupted in diabetic wounds, leading to impaired stratification and barrier function [27]. Hyperglycemia induces decreased proliferation and increased apoptosis of keratinocytes, resulting in delayed re-epithelialization and wound closure [33]. Similar to fibroblasts, keratinocytes in diabetic conditions show diminished responsiveness to growth factors such as epidermal growth factor (EGF) and keratinocyte growth factor (KGF) [24].

#### 4.1.3 Endothelial Cells

Endothelial cells are crucial for angiogenesis and the restoration of blood supply to the wound area. VEGF increases vascular permeability by affecting the expression of intercellular adhesion molecules and selectins in endothelial cells, promoting the recruitment of leukocytes to the wound bed during the inflammatory stage. VEGF significantly promotes the proliferation and migration of endothelial cells during the proliferative stage, and it stimulates the assembly of endothelial cells to facilitate the formation of the vascular lumen during the remodeling stage [34]. Under diabetic conditions, endothelial cells exhibit reduced proliferation, migration, and tube formation capacity, leading to inadequate angiogenesis in wounds [7]. Endothelial cells are susceptible to apoptosis and lose their structural integrity in a high glucose environment, resulting in angiogenesis disorders [35]. Hyperglycemia induces oxidative stress in endothelial cells, leading to cellular dysfunction and apoptosis [36]. The endothelial glycocalyx, a protective layer on the luminal surface of blood vessels, is often degraded in diabetes, leading to increased vascular permeability and inflammation [37]. In diabetes, endothelial cell dysfunction is characterized by impaired nitric oxide (NO) bioavailability attributed to reduced NO production and increased inactivation of NO by reactive oxygen species, which subsequently impairs vasodilation and blood flow regulation [38]. Under diabetic conditions, endothelial cells may undergo endothelial-to-mesenchymal transition (EndMT), contributing to fibrosis and impaired wound healing [39].

Understanding these cellular dysfunctions provides insights into potential therapeutic targets for improving diabetic wound healing. Strategies focused on restoring normal cellular functions, including growth factor therapies, cell-based treatments, and modulation of the wound microenvironment, may hold promise for enhancing wound closure in diabetic patients.

### 4.2 Altered Inflammatory Response

The inflammatory phase of wound healing is significantly dysregulated in diabetic individuals, and this is closely associated with functional alterations in various immune cells. These aberrant behaviors lead to a persistent inflammatory state, thereby impeding the normal wound healing process.

#### 4.2.1 Neutrophils

Neutrophils are the primary effector cells in acute inflammatory responses and play an important role in the early phase of healing. In diabetic wound healing, there is an initial delay in neutrophil infiltration, followed by a prolonged inflammatory response [40]. This persistent inflammation is characterized by elevated levels of pro-inflammatory cytokines such as TNF-α, IL-1β, and IL-6, which contribute to tissue damage and impair the progression to the proliferative phase of healing [15]. Neutrophils in a hyperglycemic environment show decreased phagocytic ability, increased production of reactive oxygen species (ROS), and abnormal formation of neutrophil extracellular traps (NETs) [41]. NETs have been implicated in persistent inflammation by activating the Nod-like receptor protein (NLRP) 3 inflammasome, which further exacerbates the inflammatory response and impairs healing [42]. Neutrophils also act as chemoattractants for tissue-resident cells such as macrophages and dendritic cells by releasing IL-8 and CXCL8, thereby promoting the wound healing process [43]. However, the excessive release of inflammatory cytokines and ROS, as well as neutrophil involvement and NETs, in diabetic wounds can adversely damage tissue and slow down healing.

#### 4.2.2 Macrophages

Macrophages play crucial roles in all stages of wound healing. The diabetic microenvironment causes increased myelopoiesis, resulting in elevated circulating monocytes, which enter the wound and differentiate into pro-inflammatory M1 macrophages [44]. These M1 macrophages secrete excessive pro-inflammatory cytokines (*e.g.*, TNF-α, IL-1β) and MMPs, while the production of anti-inflammatory factors (*e.g.*, IL-10) is reduced [45]. In diabetic wounds, macrophages predominantly maintain a pro-inflammatory M1 phenotype rather than transitioning to the pro-healing M2 phenotype during the later stages of wound healing, thereby perpetuating the inflammatory state [45-47]. The hyperglycemic environment also promotes the activation of the NLRP3 inflammasome in macrophages, leading to the excessive release of IL-1β and IL-18 [48, 49]. Macrophages in the diabetic environment exhibit reduced phagocytic capacity, which impairs the clearance of apoptotic cells and debris [50]. Phagocytosis triggers the transition of the macrophage phenotype from M1 to the pro-healing M2 state within the wound [51]. The phenotypic imbalance between M1 and M2 macrophages leads to a persistent inflammatory state in diabetic wounds, which subsequently obstructs the pro-healing functions of endothelial cells, keratinocytes, and fibroblasts [51]. M2 macrophages are known to secrete growth factors such as PDGF, EGF, and VEGF, which are required for tissue repair [52]. The phenotypic shift of macrophages is a crucial aspect of the transition from the inflammatory phase to the proliferative phase [52, 53]. The impaired M1/M2 macrophage transition in diabetic wounds is associated with impaired wound closure, poor angiogenesis, and decreased collagen deposition [54].

#### 4.2.3 T Cells

T cells play important roles in regulating the wound healing process. In diabetic wounds, there is an observed increase in the proportion of Th1 cells and a decrease in Th2 cells, leading to a persistent pro-inflammatory environment [55]. Research indicates that CCR4^+^ regulatory T cells (Tregs) may impair diabetic wound healing, potentially due to their inhibitory effects on inflammation by modulating cell recruitment and activation, which are critical for the early inflammatory response in the healing process [56]. In the later stages of wound healing, the balance between Tregs and Th17 cells is skewed to favor the inflammatory CD4^+^ Th17 cells [57]. γδT cells also play a particularly important role in wound healing through their production of insulin-like growth factor-1 (IGF-1) and keratinocyte growth factor (KGF), both of which are crucial for keratinocyte proliferation and wound re-epithelialization. The γδ T cells show alterations in both number and function in diabetic wounds, affecting epidermal repair and angiogenesis [58]. Patients with diabetic wounds have fewer skin-resident γδT cells compared to those with non-diabetic wounds [58]. Dendritic epidermal T cells (DETCs) constitute a vital component of the innate immune surveillance system within the skin. The quantity of DETCs diminishes in diabetic mouse models, resulting in reduced production of IGF-1 and KGF in the epidermis, which is closely associated with delayed wound healing in diabetes [59].

#### 4.2.4 Mast Cells

Mast cells play an important role in the early stages of wound healing. During the remodeling phase of wound healing, mast cells increase rapidly and accumulate in the wound, whereas the increase of mast cells is delayed in diabetic mice [60]. Mast cells and their secreted mediators participate in all stages of wound healing and contribute to the pathogenesis of chronic diabetic foot ulcers. Specifically, increased degranulation of skin mast cells occurs in diabetes, which is associated with impaired wound healing [61]. The hyperglycemic environment leads to decreased VEGF release and excessive production of pro-inflammatory mediators from mast cells [62].

#### 4.2.5 Dendritic Cells

Dendritic cells, which bridge innate and adaptive immunity, also show functional abnormalities in diabetic wounds. Under diabetic conditions, dendritic cell migration to the wound is impaired [63], thereby affecting antigen presentation. The hyperglycemic environment leads to phenotypic changes in dendritic cells, compromising their ability to activate T cells [64]. Plasmacytoid dendritic cells (pDCs) play an important role in interacting with naive CD4^+^ T cells to differentiate them into CD4^+^ Th17 cells by increasing the production of IL-6 in diabetic wounds, which is responsible for chronic T cell-mediated inflammation in diabetic wound healing [57].

These alterations in immune cells collectively contribute to the persistent inflammatory state and delayed healing in diabetic wounds. Understanding these cellular functional changes provides an important basis for developing new therapeutic strategies, such as targeting specific immune cell subsets, modulating cytokine networks, or improving cellular functions, which may improve outcomes in diabetic wound healing.

### 4.3 Complex Cellular Interactions in Diabetic Wound Healing

Diabetic wounds are a significant clinical challenge due to the impaired function of various cell types involved in the wound healing process. The interaction between these cells is crucial for effective healing, and disruptions can lead to chronic wounds that are difficult to treat.

Macrophages in diabetic wounds exhibit heightened inflammatory responses, characterized by elevated production of TNF-α and IL-1β [65]. These pro-inflammatory cytokines have been shown to substantially inhibit the proliferation and migration of fibroblasts, keratinocytes, and endothelial cells, creating a counterproductive environment for wound repair [51]. Macrophage-derived VEGF is essential for the function of endothelial cells and endothelial progenitor cells associated with neovascularization in the proliferative and tissue-regeneration phase of healing [66]. The exosomes released by endothelial cells can also influence the polarization state of macrophages, thereby affecting the wound healing process [67]. Decreased production of key growth factors secreted by M2 macrophages in diabetes, particularly TGF-β1 and PDGF, significantly impacts fibroblast proliferation, extracellular matrix synthesis, and wound contraction [5, 68]. Administering TGF-β1 to diabetic mice facilitated angiogenesis and wound healing, and balanced M1/M2 macrophage polarization in the granulation tissue [69]. Fibroblasts also secrete chemokines such as chemokine (C-C motif) ligand 2 (CCL2), which can attract macrophages to the wound site and maintain appropriate polarization states of macrophages [70]. However, in hyperglycemic conditions, this regulatory mechanism becomes disrupted, leading to persistent inflammation and impaired tissue repair [70]. In addition, NETs are excessively formed in diabetic conditions, leading to delayed wound healing and chronic inflammation. Excessive NETs significantly impairs fibroblast migration and proliferation *via* endoplasmic reticulum stress in diabetic wounds [71]. NET-derived MMP-9 and histones also damage endothelial integrity and function in wound healing [72].

### 4.4 Oxidative Stress and AGEs

Hyperglycemia-induced oxidative stress plays a significant role in the pathological process of diabetic wound healing. Oxidative stress may affect diabetic wound healing *via* skin damage, nerve damage, ischemic injury, and localized infection [73]. The overproduction of reactive oxygen species (ROS), coupled with inadequate antioxidant systems, results in oxidative stress. Hyperglycemia increases the production of superoxide anion in skin tissue *via* activation of NADPH oxidase and protein kinase C, which delays wound healing in diabetic mice [74]. Elevated glucose levels lead to increased production of ROS, overwhelming the antioxidant defenses of cells [75]. This oxidative imbalance causes cellular damage, impairs angiogenesis, and promotes inflammation [76].

Additionally, the formation of AGEs due to non-enzymatic glycation of proteins further exacerbates oxidative stress and inflammation. AGEs interact with the receptor for AGE (RAGE), triggering intracellular signaling cascades that promote the production of inflammatory mediators and impair cellular function [77]. AGEs induce activation of NADPH oxidase and excessive production of ROS by binding to receptors on the endothelial cell surface [78]. Excess oxidative stress originating from the AGE-RAGE axis can activate NF-қB signaling and impact the transcriptional activation of numerous cytokines and adhesion molecules, which are closely associated with inflammation and angiopathy in diabetic wound healing [78]. Moreover, AGEs also hinder diabetic wound healing by inhibiting the proliferation of fibroblasts and stimulating autophagy associated with M1 polarization of macrophages [79, 80].

### 4.5 Neuropathy and Vascular Complications

Diabetic neuropathy and vascular complications significantly contribute to impaired wound healing. Peripheral neuropathy leads to the loss of protective sensation, increasing the risk of trauma and wound formation [81, 82]. Autonomic neuropathy affects sweat gland function, leading to dry, fragile skin that is more prone to injury [82-84]. Vascular complications, including both macrovascular and microvascular disease, result in reduced blood flow to the wound area, compromising the delivery of oxygen, nutrients, and immune cells necessary for healing [7]. The combination of neuropathy and vascular insufficiency creates a particularly challenging environment for wound healing, often leading to the development of chronic, non-healing ulcers [85]. Endothelial progenitor cells (EPCs) play a crucial role in wound healing, accelerating the process by facilitating the formation of new blood vessels. However, in the diabetic environment, the number of EPCs is reduced, and the functions of EPCs, such as migration, adhesion, and tube formation, are often suppressed [86, 87]. EPC dysfunction leads to insufficient angiogenesis and delayed wound healing.

Understanding these pathophysiological mechanisms provides a foundation for the development of targeted therapies aimed at addressing the complex nature of impaired wound healing in diabetic patients. Future research should focus on elucidating the intricate interplay between these factors and identifying novel therapeutic targets to enhance wound healing in diabetic patients.

## 5 INNOVATIVE INTERVENTIONS FOR DIABETIC WOUND HEALING

The complex pathophysiology of diabetic wounds necessitates a multifaceted therapeutic approach. Recent advancements in biomaterials, cellular therapies, and molecular interventions have opened new avenues for treating diabetic wounds. This section explores innovative interventions that show promise in enhancing diabetic wound healing. Some clinical trials involving the above methods are listed in Table **1**.

### 5.1 Advanced Dressings and Biomaterials

Advanced dressings and biomaterials offer promising alternatives to traditional wound dressings such as cotton and gauze, which enhance the healing process and provide better outcomes. One of the key advantages of advanced wound dressings is their ability to provide a moist wound environment and protect the wound from secondary infections, which is crucial for promoting healing. Additionally, the use of biomaterials in dressings has been explored for their improved biocompatibility and ability to support cell growth and tissue repair. Another significant advantage of advanced dressings is their ability to deliver therapeutic agents directly to the wound site. Furthermore, the development of smart dressings that respond to the wound environment is an exciting area of research. These dressings can adapt to changes in the wound's pH, temperature, or moisture levels, providing a more dynamic and responsive treatment approach. These advanced dressings and biomaterials mainly consist of nanofibers, hydrogels, scaffolds, polymeric nanoparticles, liposomes, and inorganic nanoparticles (Fig. **2**).

Recently, new dressings such as hydrogels and nanofibers have received much attention for diabetic wound healing [88]. The development of advanced wound dressings and biomaterials has significantly improved diabetic wound management due to their exceptional biocompatibility and biodegradability. In a randomized clinical trial of 46 cases of diabetic foot ulcers, the complete healing rate in the group treated with an acellular flowable matrix was higher compared to the control group (86.95% *versus* 52.17% in the control group) [89]. In another randomised controlled trial, the complete healing rate in the group treated with advanced wound dressing is higher compared to the control group (85.29% *versus* 15.38% in the control group) during the trial [90]. These responsive materials can respond to the wound environment, providing optimal moisture balance and controlled release of therapeutic agents. For example, glucose-responsive hydrogels can release insulin or antimicrobial agents in response to elevated glucose levels in the wound [91]. In addition, a glucose-responsive drug-loaded metal-organic hydrogel effectively promoted angiogenesis, collagen deposition, and re-epithelialization during the wound healing process in diabetic mice [91].

What’s more, nanoparticle-based drug delivery systems, which include liposomes, polymeric nanoparticles, inorganic nanoparticles, lipid nanoparticles, nanofibrous structures, and nanohydrogel, can enhance the stability and targeted delivery of therapeutic agents [92]. Electrospun nanofiber scaffolds feature a unique porous architecture similar to the extracellular matrix structure, which promotes cell adhesion, proliferation, and migration [93]. These scaffolds can be functionalized with growth factors or antimicrobial agents to enhance wound healing [93, 94]. Self-assembling peptides (SAP) are another type of synthetic hydrogel that can form cross-linked nanofibers autonomously and change into three- dimensional porous hydrogels rapidly [95]. These biomaterials can be designed to incorporate bioactive molecules that promote angiogenesis and tissue repair, and provide a favorable microenvironment for cell migration and tissue regeneration [95]. In summary, advanced wound dressings and biomaterials offer several advantages over traditional methods in the treatment of diabetic wounds, representing a significant advancement in wound care and offering hope for improved management.

### 5.2 Growth Factor Therapies

Growth factors such as VEGF, EGF, FGF, and PDGF play crucial roles in angiogenesis, cell proliferation, and migration, which are essential processes in wound healing. Their targeted delivery has shown promising results. Autologous platelet-rich plasma (PRP) provides a concentrated source of growth factors, promoting tissue regeneration and angiogenesis. Recent studies have shown improved wound closure rates with PRP treatment in diabetic foot ulcers [96, 97]. The wound healing rate of patients in the PRP treatment group was higher than that in the control group, and the healing time was also faster than that in the control group (31.40±4.47 days versus 43.20±5.03 days in the control group). Topical application of growth factors, such as PDGF-BB, TGF, EGF, VEGF, KGF, IGF, and FGF, has demonstrated efficacy in accelerating diabetic wound healing [98]. The average wound healing rate is 94% for the treatment of CT-102, which is derived from homologous platelets and contains numerous growth factors, compared to 73% for the placebo group [99]. Innovative delivery systems, including nanoparticles and hydrogels, have been developed to enhance the stability and sustained release of these factors [100]. In addition, a combination of growth factors such as VEGF and PDGF has shown enhanced angiogenesis and wound closure in diabetic mice [101].

### 5.3 Cell-based Therapies

Stem cell-based interventions offer regenerative potential for diabetic wounds. Diabetic wounds are characterized by impaired healing processes, including cellular dysfunction, reduced angiogenesis, chronic inflammation, and neuropathy. Among cell-based therapies, mesenchymal stem cells (MSCs) have demonstrated potential in overcoming these challenges through various mechanisms. MSCs derived from various sources, such as bone marrow, adipose tissue, and umbilical cord, have shown promise in enhancing diabetic wound healing. Their paracrine effects, immunomodulatory properties, and differentiation potential contribute to improved wound closure and tissue regeneration (Fig. **3**) [102, 103]. A growing amount of evidence shows that the paracrine effects, which manifest relatively early, play a crucial role in MSC-based therapy. Recent studies have demonstrated that MSCs release extracellular vesicles, growth factors, cytokines, and chemokines that modulate the local inflammatory environment, stimulate angiogenesis, promote fibroblast activity, and enhance re-epithelialization [104, 105].

Autologous epidermal stem cell therapies, such as cultured epidermal autografts, have demonstrated efficacy in treating chronic diabetic ulcers by enhancing re-epithelialization [106]. Moreover, EPC therapy aims to improve wound vascularization. Recent studies have explored methods to enhance EPC function and their ability to home to wound sites in diabetic conditions. Growth differentiation factor 11 (GDF11) enhances the process of diabetic wound healing by stimulating the mobilization of EPCs and neovascularization, which is mediated by the HIF-1ɑ-VEGF/SDF-1ɑ pathway [107]. Adipose-derived stem cells, autologous bone marrow-derived cells, and mesenchymal stem cells have been used for stem cell-based treatment of diabetic foot ulcers [103]. Treatment with autologous bone marrow-derived mononuclear cells leads to a considerable increase in the vascular network in diabetic patients according to the assessment of digital subtraction angiography [108]. Patients with diabetic foot ulcers who receive bone marrow mesenchymal stromal cells therapy exhibit a higher wound closure rate and enhanced skin regeneration compared to those receiving conventional treatments [109]. The wound size decreases from 7.26±1.41 cm^2^ to 2±0.98 cm^2^ (*p* < 0.001) at 12 weeks in the diabetic foot ulcer group treated with bone marrow-derived mesenchymal stem cells [110].

Despite these promising results, clinical outcomes remain variable, underscoring the need for combined approaches that address multiple aspects of wound healing pathology simultaneously. Cell therapy alone is insufficient to completely repair diabetic ulcers, which may be attributed to the decreased survival rate, reduced homing ability, and compromised differentiation potential of transplanted cells. Future directions include optimizing cell delivery methods, developing bioactive scaffolds that enhance the function of transplanted cells, and exploring combined approaches.

### 5.4 Gene Therapy Approaches

Gene therapy presents a promising avenue for addressing the underlying molecular and cellular deficits associated with diabetic wound healing. This therapeutic approach enables targeted intervention by delivering specific genes that can enhance the wound healing process. Viral and non-viral vectors for the local delivery of growth factor genes such as VEGF and PDGF have shown promise in enhancing angiogenesis and tissue repair in diabetic wounds [111]. *in vivo* gene therapy targeting inflammatory pericytes *via* an adenovirus vector carrying the oxidation resistance protein 1 gene can inhibit the oxidative stress and apoptosis of pericytes, thereby improving diabetic wound healing [112]. Patients with diabetic foot ulcers are randomly assigned to the Ad5PDGF-B treatment group and control group, and the group administered with Ad5PDGF-B (GAM501) locally exhibits a higher incidence of complete closure compared to the control group (41% *versus* 31%) [113]. In addition, gene-editing tools offer potential for correcting genetic factors contributing to impaired wound healing in diabetes. N-myc downregulated gene 2 (Ndrg2) knockout murine dendritic cells exhibit strong pro-angiogenic and regenerative capacity, which accelerates the chronic wound healing [114].

Targeting specific microRNAs involved in wound healing pathways has also emerged as a novel approach (Table **2**). For instance, inhibition of miR-26a and local injection of miR-31-5p have been shown to promote angiogenesis and accelerate wound healing in diabetic mice [115-117]. Upregulation of miR-146a expression promotes wound healing in diabetic mice by inhibiting its downstream pro-inflammatory target genes such as IRAK1 and TRAF6 [118]. Inhibition of miR-155 has been shown to promote re-epithelialization and inhibit the inflammatory situation [119, 120]. Downregulation of miR-29a or miR-29b may improve collagen synthesis and ECM remodeling [121-123]. Inhibition of miR-210, miR-200b, and miR-15b can promote angiogenesis [124-129]. Local replenishment of miR-132 in the wounds of db/db mice enhances wound healing by promoting keratinocyte proliferation [130, 131].

Recent advances have focused on combining different treatment modalities to achieve synergistic effects and overcome the limitations of single-agent therapies. For example, a recent study demonstrated the efficacy of hydrogel-based gene-activated materials containing plasmid DNA encoding VEGF in accelerating wound healing in patients with diabetic foot ulcers [132, 133]. The combination of an appropriate scaffold material with gene therapy enables prolonged gene expression and creates a favorable microenvironment for tissue regeneration [132, 134].

## CONCLUSION

In recent years, the field of diabetic wound healing has undergone significant advancements, largely attributed to a deeper understanding of the complex pathophysiology and the development of innovative therapeutic approaches. These advancements span various areas, including advanced dressings and biomaterials, cellular therapies, and molecular interventions.

The complex nature of diabetic wound healing necessitates a comprehensive and integrated treatment approach. Combination therapies that target multiple pathways and incorporate various treatment modalities appear to offer the most promise for improving outcomes. The integration of advanced wound dressings with growth factor or cell-based therapies can provide synergistic effects, addressing both the local wound environment and underlying cellular dysfunction. Moreover, the combination of gene therapy approaches with nanotechnology-based delivery systems can enhance the efficacy and specificity of molecular interventions. Furthermore, incorporating systemic treatments to address metabolic abnormalities, in conjunction with local wound therapies, is essential for the comprehensive management of diabetic wounds.

Despite the promising results of advanced therapies, important safety considerations must be addressed, particularly regarding the potential oncogenic risks associated with growth factors, cell therapies, and gene therapies. Growth factors such as VEGF, EGF, and PDGF play crucial roles in tumor development and progression [135]. Similarly, the immunomodulatory and pro-angiogenic properties of stem cells, while beneficial for wound healing, are associated with the risks of malignant transformation and cancer induction [136]. Gene therapies, especially those involving viral vectors, carry risks of insertional mutagenesis and unintended activation of oncogenes [137]. Therefore, careful screening and risk assessment are essential before initiating these advanced therapies. Thorough evaluation of patients, especially elderly individuals, for any history of malignancy or precancerous conditions is crucial. Regular follow-up and monitoring during and after treatment are also necessary to detect any adverse effects or abnormal tissue growth. To mitigate these risks, several strategies can be employed, including local administration, transient genetic modifications, and non-viral gene delivery.

In conclusion, although significant progress has been made in the field of diabetic wound healing, challenges remain in translating these advancements into widely accessible and cost-effective treatments. Future research should focus on optimizing combination therapies, developing personalized treatment algorithms, and conducting large-scale clinical trials to validate the efficacy and safety of novel interventions.

## Figures and Tables

**Fig. (1):**
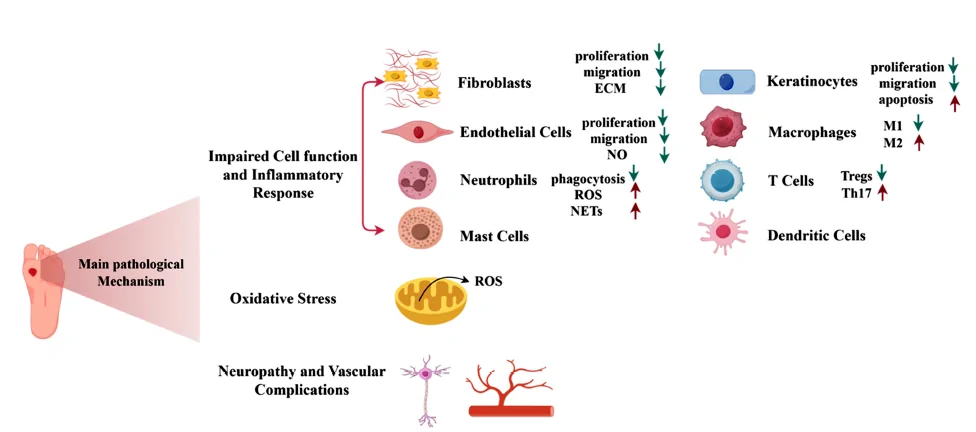
The main pathological mechanisms of diabetic wound healing. The pathophysiology of diabetic wounds involves impaired cellular function and inflammatory responses, oxidative stress, neuropathy, and vascular complications.

**Fig. (2):**
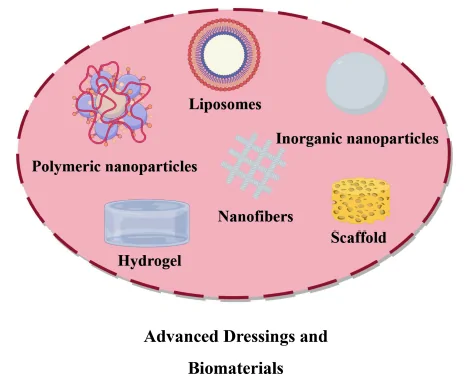
Schematic representation of advanced dressings and biomaterials for diabetic wound healing (created with Figdraw). The major categories include nanofibers, hydrogels, scaffolds, polymeric nanoparticles, liposomes, and inorganic nanoparticles.

**Fig. (3):**
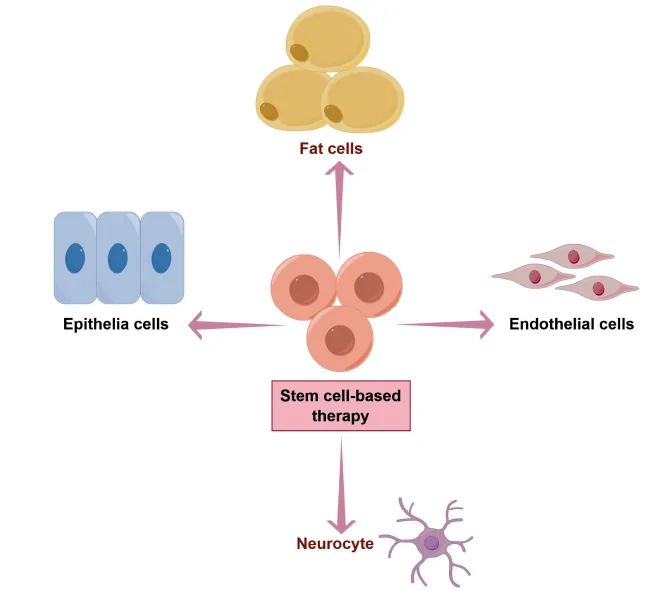
Cell-based therapies for diabetic wound healing. The differentiation potential of stem cells contributes to improved wound closure and tissue regeneration.

**Table 1:** Clinical trials of interventions for diabetic wound healing.

**Therapeutic Approaches**	**Intervention Type**	**Study Design**	**Key Findings**	**References**
Advanced Dressings	An acellular flowable matrix	A randomized clinical trial	Higher complete healing rate (86.95% *vs* 52.17% controls).	[89]
	Advanced wound dressing	A randomized controlled trial	Higher complete healing rate (85.29% *vs* 15.38% controls).	[90]
Growth Factor Therapies	Platelet-rich plasma (PRP)	Comparative study	PRP significantly improved wound healing time (31.40±4.47 *vs* 43.20±5.03 days).	[96]
	CT-102 is derived from homologous platelets and contains many growth factors	Randomized prospective double-blind trial	Average percent reduction in ulcer area at 20 wk (94% *vs* 73% control).	[99]
Cell-based Therapies	Bone marrow-derived mononuclear cell	Clinical trial	A considerable increase in the vascular network.	[108]
	Bone marrow mesenchymal stromal cells	Clinical trial	Higher wound closure rate and enhanced skin regeneration.	[109]
	Autologous bone marrow-derived mesenchymal stem cells	Randomized controlled trial	The wound size decreases from 7.26±1.41 cm^2^ to 2±0.98 cm^2^ (*p* < 0.001) at 12 weeks.	[110]
Gene Therapy	Local Ad5PDGF-B (GAM501) delivery	Clinical trial	Higher complete healing rate (41% *vs* 31% controls).	[113]

**Table 2:** Targeting specific microRNAs for the therapy of diabetic wounds.

**miRNAs**	**Role in Diabetic Wound Healing**	**Target Genes/Pathways**	**Potential Therapeutic Strategy**	**References**
miR-26a	Inhibits angiogenesis and wound closure	SMAD1, VEGF-A	Inhibition of miR-26a	[115, 117]
miR-146a	Promotes wound healing by inhibiting the inflammatory response	IRAK1, TRAF6	Upregulation of miR-146a	[118]
miR-155	Promotes inflammation and inhibits re-epithelialization	SOCS1, SHIP1	Inhibition of miR-155	[119, 120]
miR-29a	Impairs collagen synthesis and ECM remodeling	TAB-1	Downregulation of miR-29a	[121]
miR-29b	Impairs collagen synthesis and ECM remodeling	COL1A1, COL3A1	Downregulation of miR-29b	[122, 123]
miR-210	Inhibits angiogenesis under hypoxic conditions	EFNA3, PTP1B	Inhibition of miR-210	[124-126]
miR-200b	Inhibits angiogenesis and endothelial function	ETS1, GATA2, VEGFR2	Inhibition of miR-200b	[127]
miR-15b	Impairs wound healing by inhibiting angiogenesis	VEGF, BCL-2	Inhibition of miR-15b	[128, 129]
miR-132	Enhances wound healing by promoting keratinocyte proliferation	HB-EGF	Upregulation of miR-132	[130, 131]

## LIST OF ABBREVIATIONS

AGEs: Advanced Glycation End-products
MMPs: Matrix Metalloproteinases
PDGF: Platelet-derived Growth Factor
TIMPs: Tissue Inhibitors of Matrix Metalloproteinases
TGF-β: Transforming Growth Factor Beta
VEGF: Vascular Endothelial Growth Factor
